# Editorial Note: Enterprise digitization and marine economic performance: An empirical study of listed enterprises in China’s maritime economy

**DOI:** 10.1371/journal.pone.0359695

**Published:** 2026-10-12

**Authors:** The PLOS One Editors

The *PLOS One* Editors issue an Editorial Note for this article [1] to notify readers of the following:

- The articles cited as References 12, 21, and 55 do not appear to support the corresponding cited statements.
- The article cited as Reference 56 could not be retrieved by the Editors.

The first author provided information to explain the relevance of References 12, 21, and 55, but the editors do not consider this issue resolved.

The first author acknowledged that that Reference 56 was introduced as an unintentional error and should refer to Reference 55.

In addition to the above, there is an error in Reference 16. The correct reference is: Li Jinchang, Lian Ganghui, and Xu Songting. An empirical study on digitization-driven greening as a solution to corporate green transformation under the “dual-carbon” vision. Journal of Quantitative and Technological Economics, 2023, 40(9): 27–49
